# Editorial: Dialogues in Music Therapy and Music Neuroscience: Collaborative Understanding Driving Clinical Advances

**DOI:** 10.3389/fnhum.2016.00585

**Published:** 2016-11-22

**Authors:** Julian O'Kelly, Jörg C. Fachner, Mari Tervaniemi

**Affiliations:** ^1^Unit for Social and Community Psychiatry, WHO Collaborating Centre for Mental Health Services DevelopmentLondon, UK; ^2^Research, Royal Hospital for Neuro-DisabilityLondon, UK; ^3^Music and Performing Arts, Anglia Ruskin UniversityCambridge, UK; ^4^Cicero Learning, University of HelsinkiHelsinki, Finland

**Keywords:** music therapy, neuroscience methods, neuro-rehabilitation, neuro-degenerative diseases, psychoimmunology, EEG/ERP, fMRI, neurophysiology

Over 30 years of neuroscientific investigations of music perception and cognition have developed an understanding of music involving and supporting global brain processing in virtually every sphere of human activity (Levitin and Tirovolas, 2009; Särkämö et al., 2013). Increasingly sophisticated neuroimaging technology is able to provide objective biomedical evidence of musical activity supporting neuroplasticity (Münte et al., 2002; Pantev and Herholz, 2011), which has been shown as underpinning recovery and rehabilitation in stroke (Schlaug et al., 2009; Särkämö et al., 2014). Furthermore, the therapeutic potential of musical activity has been evidenced by neuroscience methods in relation to effects between common areas of processing between speech, memory, attention and motor activity (Schlaug et al., 2009; Besson et al., 2011; Patel, 2011), in how it influences arousal (Pelletier, 2004; O'Kelly et al., 2013), and through the modulation of wide ranging neurochemical activity involved in stress, immunity, social affiliation, and reward (Chanda and Levitin, 2013; Fancourt et al., 2014).

The effective use of music with clinical populations by credentialed music therapists is also an area of increasingly robust research activity, as evidenced by recent Cochrane reviews of music therapy with autism (Geretsegger et al., 2014), depression (Maratos et al., 2008) schizophrenia (Mössler et al., 2011), and acquired brain injury (Bradt et al., 2010). Various interventions designed to address functional deficits and health care needs have been developed (e.g., Thaut and Hoemberg, 2014), alongside valid and reliable scales sensitive to the effects of music in assessment and treatment with diverse clinical populations, including disorders of consciousness (Magee et al., 2014), parent–child relationships (Jacobsen and McKinney, 2015), and neurodegenerative conditions such as dementia (McDermott et al., 2014) and Huntington's disease (O'Kelly and Bodak, 2016). Historically, music therapy has drawn its evidence base from a number of contrasting theoretical frameworks, wherein an abundance of heterogeneous, sometimes contradictory theoretical approaches are hard to generalize to a wider multidisciplinary and international milieu (Hillecke et al., 2005). Clinicians are now turning to neuroscience, which offers a unifying knowledge base and frame of reference to understand and measure therapeutic interventions from a biomedical perspective.

Neuroscience methods offer exciting opportunities to understand the effects and mechanisms involved in music therapy practice through (i) *in situ* studies, where measures are used during music therapy sessions to explore underlying neurological processes, (ii) *empirical comparisons* where neuroimaging and neurological measures provide biomarkers of general changes in brain processes pre and post interventions, and (iii) *approximations*, where methods are focussed on the effects of specific musical features, and findings explored to identify brain based action mechanisms in the music therapy process (Fachner, 2016).

Whilst music therapy is benefiting from neuroscience collaborations, neuroscience is becoming more enriched by learning about the neural effects of “real world” clinical applications in music therapy. Not only do neuroscientific imaging methods provide biomarking evidence for the efficacy of music therapy interventions, they also offer important tools to describe time-locked interactive therapy processes, feeding into the emerging field of social neuroscience. Music therapy is bound to the process of creating and experiencing music together in improvisation, listening and reflection. Thus the situated cognition and experience of music developing over time and in differing contexts is of interest in time series data (Fachner, 2014, 2016).

This research topic developed as a consequence of the editors shared commitment to promoting fruitful dialogues between music therapists, psychologists, neuroscientists, and other medical professionals, at a time where these professions are increasingly sharing the same platforms at conferences (e.g., Luck and Brabant, 2013; Bigand and Tillmann, 2015; O'Kelly et al., 2014), and in collaborative research topics such as this, and its predecessor of a similar theme (Tervaniemi, 2014, for music and brain plasticity; Särkämö et al., 2016 for music in neurorehabilitation). Our research topic features the work of 115 authors in 18 papers across the titles Frontiers in Human Neuroscience and Frontiers in Auditory Cognitive Neuroscience. Whilst the majority of papers detail music therapy and neuroscience collaborations with specific clinical populations, two further areas are covered (i) commentary on the challenges and opportunities of music therapy and neuroscience collaborations, and (ii) studies with healthy populations offering both insights into how we process music and transferrable lessons for music therapy.

A diverse range of clinical populations are covered by the topic, reflecting the many areas of health care where music therapy and neuroscience collaborations are providing important new understandings. Two areas of stoke rehabilitation are detailed in the topic. Street et al. outline feasibility, efficacy, and patient experience of a music therapy treatment protocol aimed at promoting measurable changes in upper limb function in hemiparetic stroke patients. The authors detail the use a neurologic music therapy (NMT) technique designed for this purpose: *Therapeutic Instrumental Music Performance*. Similarly, Cortese et al. explore the effectiveness of “Melodic-Rhythmic Therapy” in the treatment of aphasia with six stroke patients. Bukowska et al. combine several NMT techniques (“*Therapeutic Instrumental Music Performance*,” “*Rhythmic Auditory Stimulation,” and “Pattern Sensory Enhancement”*) in a pilot study examining mobility and stability with Parkinsons patients. Using a range of novel methods including 3D Movement Analysis, the authors demonstrate significant improvement in the majority of the spatiotemporal gait parameters in the experimental group (*n*:30) compared to the control group (*n*:25).

Neurological populations feature in several other studies in the topic, such as Baker et al.'s exploration of flow and meaningfulness using songwriting with those with traumatic brain injuries or spinal cord injury. Whilst they found the intervention was positively associated with well-being outcome in the 10 participants, they also observed that those who found the songwriting process had strong personal meaning, experienced increased anxiety and depression in the process of accepting their emotions. Steinhoff et al. (2015) detail a pilot music therapy study with five individuals in an unresponsive wakefulness state (or “vegetative state”) using Positron Emission Tomography to measure changes in brain activity, finding increases in tracer uptake across the frontal, hippocampal, and cerebellar region of the brain of four patients receiving music therapy for 5 weeks. Krick et al. (2015) use structural brain scanning (magnetic resonance imaging, MRI) methods to examine the effects of the Heidelberg model of music therapy on tinnitus at a cortical level. The authors found increases in gray matter volume of a range of brain areas dedicated to auditory processing concurrently with decreased symptoms. Finally, neuro-developmental issues in autistic spectrum disorder (ASD) are addressed in a study of the effect of sung speech on socio-communicative responsiveness by Paul et al. Using an adapted single subject design with three autistic children, the authors concluded sung directives may play a useful role in engaging children with ASD serving as an effective intervention for promoting socio-communicative responsiveness.

Ramirez et al. (2015) detail a pilot study using a novel musical EEG neurofeedback system to treat depression in the elderly. The authors found an average of 17% improvements on depression scores (BDI), concurrent with significant decreases of relative alpha activity in their left frontal lobe (*p* = 0.00008) in subjects receiving the intervention. As with all the studies in the topic featuring pilot level data, the small sample (*n*:10) in the study suggests findings must be cautiously interpreted, but points to the potential benefits of this novel technology. Linnemann et al. detail a study of voluntary (not therapist initiated) music listening with 30 female participants with fibromyalgia syndrome, which is characterized by chronic pain. Whilst neuro-chemical measures of stress response (cortisol and alpha-amylase) did not indicate significant effects, significantly improved perceived control over pain was observed using VAS type Ecological Momentary Assessment Items. The final study with a clinical population was provided by Fritz et al., in their study of the psychological effects of listening to self-made music during a prior musical feedback intervention with 22 polydrug abusers. The study design compared scores range of scales (e.g., PANAS) completed after listening to pre-recorded drum and base music or listening back to music co-created with other participants on exercise machines (“Jymmin”) capable of modulating musical sounds, producing a similar, but original co-created music. They found a positive effect of listening to the recording of joint music making on self-efficacy, mood, and a readiness to engage socially, proposing participants were influenced by “recapitulating intense pleasant social interactions during the Jymmin intervention” (p. 1).

As detailed, a range of review and commentary papers explore the challenges and opportunities afforded by neuroscience and music therapy collaborations. Magee and Stewart frame this discussion by exploring existing and potential collaborations, whilst highlighting the misconceptions from both parties that may impede further expansion of the field. In a similar vein, Hunt comments on the boundaries of research methods employed in the neurosciences with regard to capturing inter-subjective, holistic experiences in music therapy, highlighting the potential of emerging technologies providing methods for delivering clinically relevant information for music therapists. Further to providing an overview of our neuroscientific understanding of auditory processing in premature infants, Shoemark et al. build the case for music-based interventions, including a hypothetical vignette from their shared clinical experience. Similarly Moore and Hanson-Abromeit review the neuroscience of emotional regulation development in childhood to frame the rationale for “Musical Contour Regulation Facilitation,” an interactive intervention for emotional regulation. Finally Sachs et al. provide a systematic review of the “Pleasures of Sad Music,” concluding that such pleasures exist where music is perceived as non-threatening, is aesthetically pleasing, and where it produces psychological benefits such as mood regulation. The authors continue by exploring the neural mechanisms involved in producing sadness which can also induce a positive affective states, with implications for informing music therapy work in this field.

A range of research papers explore the therapeutic potential of music through neuroscientific investigations with non-clinical participants. Here, the emotional effects of music are explored from a range of perspectives. Psychologists Sharman and Dingle explore the conception that listening to music from the extreme metal genre might have a causal relationship with anger behaviors. Thirty-nine extreme metal fans were tested for heart rate and positive/negative feelings of effect on the PANAS scale after an “anger induction” followed by listening to 10 min of either preferred music or silence. Contrary to expectations, participants reacted to their preferred extreme music with stabilized heart rate and positive emotions. The role of music listening in emotional regulation receives further attention from Carlson et al., who investigated relationships between music listening behaviors (“Discharge” or “Diversion”) and gender, levels of depression, anxiety and neuroticism in a large non-clinical sample (*n*:123). Interestingly, in the context of Sharman and Dingles findings, Carlson et al. found on psychological scales (MADRS, BFQ, and HADS-A) that Discharge (using music to express negative emotions), was related to increased anxiety and Neuroticism, particularly in males. However, comparisons can only be made cautiously given the different samples, methods and range of genres involved in Carlson's study. Carlson et al. also present brain imaging (functional magnetic resonance imaging, fMRI) findings highlighting decreases in medial prefrontal cortex activity in high Discharge males, with increases for females preferring music listening for Diversion, exploring these findings in relation to the neurological and psychological impact of maladaptive listening practices.

Focussing on more active music making in non-clinical populations, the effects of improvised and pre-composed choral singing on experiences of flow, engagement, and neurobiological measures of social affiliation and arousal (oxytocin and adrenocorticotropic hormone/ACTH) were investigated by Keeler et al. Significant increases on a validated measure of flow for both singing conditions were observed concurrently with decreases in ACTH, significantly for pre-composed music. Whilst the small sample of one choral quartet with a limited range of material indicates caution, the authors propose group singing as effective in reducing stress and arousal, highlighting the importance of flow states in this process.

In setting up this research topic, we aimed to investigate the following question from different viewpoints: what can we understand about the musical, therapeutic, relational, or creative processes in music therapy from a neuroscience perspective, and how can this perspective advance music therapy practice? Moreover, we aimed at introducing various experimental approaches designed recently in order to investigate the efficacy and underlying principles of music therapy. As we hoped, authors working with those with acquired, developmental or neurodegenerative neurological and psychiatric conditions submitted empirical research, systematic reviews, and case studies adopting neuroscientific methods. Furthermore, the richness, challenges, and potentials of this field have been explored in commentary, position statement, and theoretical papers. Though, convergent thinking and research activity this volume illustrates how much music therapy and neuroscience have to learn from each other. The authors wish to thank all authors, peer reviewers, and participants in the research featured here for the important contribution to this evolving field they have made in this topic. The papers featured show the great potential for more important and synergistic collaborations to benefit the wellbeing of both clinical populations, and those interested in harnessing the therapeutic power of music in their everyday lives.

## Author contributions

JO: drafted the first version of the editorial, MT and JF: revised the first draft and made contributions from their areas of expertise.

### Conflict of interest statement

The authors declare that the research was conducted in the absence of any commercial or financial relationships that could be construed as a potential conflict of interest.
